# Work-related musculoskeletal disorders among Nigerian Physiotherapists

**DOI:** 10.1186/1471-2474-9-112

**Published:** 2008-08-18

**Authors:** Babatunde OA Adegoke, Ashiyat K Akodu, Adewale L Oyeyemi

**Affiliations:** 1 Physiotherapy Department, College of Medicine, University of Ibadan, Ibadan, Nigeria

## Abstract

**Background:**

Physiotherapists are known to be prone to Work- related musculoskeletal disorders (WRMDs) but its prevalence among physiotherapists in Nigeria has not been reported. This study investigated the prevalence and work factors of WRMDs among physiotherapists in Nigeria.

**Methods:**

A cross- sectional survey was administered to physiotherapists in different parts of Nigeria using a 2- part questionnaire with items adopted from questionnaires used for similar studies around the world. Two hundred and seventeen copies of the questionnaire were distributed for self administration but 126 physiotherapists returned completed surveys for a 58.1% response. The data were analyzed using SPPS version 10 at alpha level of 0.05. Descriptive statistics of frequency and percentages and inferential statistics of *x*^2 ^were used as appropriate for data analysis.

**Results:**

Reported 12- month prevalence of WRMDs among Nigerian physiotherapists was 91.3%. Prevalence of WRMDs was significantly higher in female physiotherapists (p = 0.007) and those with lower body mass index (p = 0.045). The low back (69.8%) was the most commonly affected body part, followed by the neck (34.1%). Fifty percent of the physiotherapists first experienced their WRMDs within five years of graduation and the highest prevalence (61.7%) was found among physiotherapists younger than 30 years. Treating large number of patients in a day was cited by most (83.5%) of the respondents as the most important work factor for their WRMDs. The most commonly adopted coping strategy identified was for the therapists to modify their position and/or the patient's position (64.3%). Majority of the respondents (87.0%) did not leave the profession but 62.6% changed and/or modified their treatment because of their WRMDs.

**Conclusion:**

The prevalence of WRMDs among physiotherapists in Nigeria is higher than most values reported for their counterparts around the world. The coping strategies and work factors of WRMDs among Nigerian physiotherapists are mostly similar to those of their counterparts elsewhere.

## Background

Musculoskeletal disorders have been described as the most notorious and common causes of severe long-term pain and physical disability that affect hundreds of millions of people across the world [1]. In the work place, the health care professionals are vulnerable to sustaining musculoskeletal disorders during the course of their work routine [2-6]. Salisk and Ozkan [7] defined WRMDs among physiotherapists as musculoskeletal injuries that result from a work-related event and several studies [4-9] have documented that work-related musculoskeletal disorders (WRMDs) are frequently experienced by physiotherapists. Literature has indeed suggested that physiotherapists are particularly susceptible to WRMDs because of the nature of their profession which is often repetitive, labour intensive and involving direct contact with patients [5,8,10]. However, Lotters et al [11] emphasized the complexity of attributing musculoskeletal disorders to work while Palmer and Smedley [12] submitted that work only partly contributes to the occurrence of musculoskeletal disorders.

The life time prevalence of WRMDs among physiotherapists has been reported to be 68% in the United Kingdom [13], 55% [14] and 91% [8] in Australia, and 85% in Turkey [7]. Low back pain is the most common WRMD among physiotherapists [5-8,14] with career and annual prevalence of low back pain among physiotherapists in the United Kingdom being reported as 68% and 58% respectively [13]. In the United States, prevalence of low back pain among physiotherapists ranged from 45% [5] to 62% [6]. Mierzejewski and Kumar [9] found the prevalence of low back pain in Canada to be 49%, while Shehab et al [15] reported a 70% prevalence of low back pain in Kuwait.

Some research indicates that WRMDs among physiotherapists may be age related and also associated with professional years of experience. Bork et al [5] indicated that physiotherapists aged more than 50 years had the lowest prevalence of WRMDs, while others [7,9,13,16] reported that most physiotherapists first developed symptoms before the age of 30 years and that majority of these initial episodes occurred within five years after graduation. The physically demanding nature of the physiotherapy profession may contribute to the occurrence of WRMDs and result in a high prevalence. Elements of physiotherapy practice which have been suggested as risk factors include: treatments which demand repetitive movements or continuous bending, lifting/transferring dependent patients, responding to unanticipated or sudden movements by patients, performing manual therapy, restricted work place, understaffing, age and sex [8,10,14]. Scientific literature from various parts of the world has also reported significant association between occupational risk factors involving high repetition rates, excessive forces, and awkward postures and musculoskeletal disorders [17].

It has been opined that the cultural values of physical therapists may make it difficult for practitioners to avoid the risks of WRMDS during their work [18]. Since these cultural values are generic and unique to physiotherapy, Nigerian physiotherapists are expected to be part of this picture despite the difference in contextual practice settings. However, little seems to be known about the occupational hazards of physiotherapy practice in Nigeria, despite the wealth of information on WRMDs among physiotherapists around the world. We speculated that investigating the prevalence and work factors of work-related musculoskeletal disorders among physiotherapists in an underserved health system as Nigeria may present a different picture from what obtains in the advanced countries of the world. This study therefore investigated the 12- month prevalence and work factors of WRMDs among physiotherapists in Nigeria.

## Methods

Approval of the University of Ibadan/University College Hospital joint Institutional Review Committee on human research was obtained before the commencement of the study. Participants in the study were physiotherapists practicing in the 26 accredited secondary and tertiary health institutions in the six geopolitical zones and federal capital territory of Nigeria [19]. The respondents were neither interns nor on administrative duties at the time of the study. Two hundred and seventeen copies of a respondent administered questionnaire and informed consent forms were posted to physiotherapists working in the accredited health institutions by surface mail. A letter of introduction explaining the purpose of the study was attached to the questionnaire. Pre- addressed stamped envelopes with the address of the corresponding authour were also included in the package sent to each hospital. After two weeks, reminders were sent to the respondents while another reminder was sent two weeks after the first one. The questionnaire was returned by only those who agreed to participate in the study.

The questionnaire for this study was based on previous published surveys [8,14] but adapted for use among Nigerians. The questionnaire had two sections and contained 27 questions (see Additional file 1). Section A of the questionnaire sought information on the demographic characteristics of the respondents, their years of experience, work setting, work status and whether they have had ergonomic training or not. Its section B contained items on WRMDs, work factors and coping strategies. Respondents were asked whether they had experienced WRMDs that we defined as discomfort, injuries or pain due to their work and lasting more than three days in the last 12 months in any part of the body [14]. Respondents who indicated experience of WRMDs symptoms in any of the body areas were asked to choose the areas of disorder that they considered as the most significant and asked further questions about their disorder. Previous studies have shown the Nordic questionnaire to be valid for assessing WRMDs [20,21]

The data for this study were analyzed using SPSS version 10 with the alpha level set at 0.05. Items that represented continuous variables such as age and years of experience as a physiotherapist were converted into categorical variables in accordance with cut-offs identified in previous related studies [7,8,11]. The cut-off for Body Mass Index (BMI) was however based on the WHO (2000) classification [22] for normal weight, overweight and obesity. Descriptive statistics of percentages and inferential statistics of χ^2 ^were used as appropriate. Fisher's exact tests were performed in cases where there were fewer than 5 expected counts in a cell. Alpha level was set at 0.05.

## Results

Two hundred and seventeen questionnaires were distributed but only 126 were returned, thus giving a percentage response of 58.1%. The 126 respondents comprising (80) 63.5% males and (46) 36.5% females had a mean age of 33.7 ± 6.8 years and body mass index (BMI) of 24.1 ± 3.5 kg/m^2^. Seventy one (56.3%) of the respondents were working in secondary health institutions while 122 (96.8%) were working full-time. Table 1 shows the detailed demographic characteristics of the respondents.

**Table 1:** Socio-demographic characteristics of participants

Characteristics	
Age (yrs) (n = 126)	
Mean (SD)	33.7 (6.8)
Range	22–57
Height (m) (n = 126)	
Mean (SD)	1.68 (0.09)
Range	1.45–1.92
Weight (Kg) (n = 126)	
Mean (SD)	68.1 (10.3)
Range	50–100
BMI* (kg/m2) (n = 126)	
Mean (SD)	24.1 (3.5)
Range	18.84–42.42
Years of PT Experience (yrs) (n = 126)	
Mean (SD)	8.43 (6.1)
Range	1–32
Gender (n = 126)	
Female	46 (36.5%)
Male	80 (63.5%)
Work Status (n = 126)	
Full Time	122 (96.8%)
Part Time	4 (3.2%)
Work Setting (n = 126)	
Tertiary	54 (42.9%)
Secondary	71 (56.3%)
No Response	1 (0.8%)
Postgraduate Training (n = 126)	
Yes	32 (25.4%)
No	94 (74.6%)
Ergonomic Training (n = 126)	
Yes	70 (55.6%)
No	47 (37.3%)
No Response	9 (7.1%)

Values are means (SD), range or percentages.

*- BMI: Body Mass Index

### Prevalence

One hundred and fifteen physiotherapists (91.3%) reported experiencing WRMDs during the 12 months preceding the study. The 12- month prevalence of WRMDs in different body parts compared to the findings of previous similar studies is presented in Table 2. The low back was the most common site of disorders (69.8%) while the elbow joint (5.6%) was the least affected body part.

**Table 2:** Comparison of 12-month prevalence by body parts from different studies.

Body areas (n)	Adegoke et al Nigeria (%)	Glover et al [11] United Kingdom (%)	West and Gardner [12] Australia (%)
Low Back (88)	69.8	37.2	22.0
Neck (43)	31.1	25.7	20.0
Shoulders (28)	22.2	14.8	10.0
Wrists/Hands (26)	20.6	12.5	14.0
Knees (20)	15.9	7.8	3.0
Upper Back (18)	14.3	18.4	11.0
Thumbs (14)	11.1	17.8	-
Ankles/Feet (12)	9.5	4.1	2.0
Hips/Thighs (8)	6.3	4.8	3.0
Elbow/Forearm (7)	5.6	5.5	3.3

Table 3 presents the respondents' characteristics. Gender (p = 0.007) and BMI (p = 0.04) were the only factors that differed between those who did or did not report a WRMD.

**Table 3:** Prevalence of WRMDs according to demographic characteristics.

Characteristics	WRMD		No WRMD		Chi Statistics
			
	n	(%)	n	(%)	
Age Group (yrs)					
< 30	44	(93.6)	3	(6.4)	
> 30	71	(89.9)	8	(10.1)	0.52 (p = 0.536)
BMI Group					
18.5–24.9	88	(94.6)	5	(5.4)	
25.0–29.9	22	(84.6)	4	(15.4)	
> 30	5	(71.4)	2	(28.6)	6.22 (p = 0.045)*
Years of PT experience (yrs)					
1–5	43	(87.8)	6	(12.2)	
6–15	54	(94.7)	3	(5.3)	
> 16	18	(90.0)	2	(10.0)	1.67 (p = 0.436)
Gender					
Female	46	(100)	0	(0.0)	
Male	69	(86.3)	11	(13.8)	6.93 (p = 0.007)*
Work Status					
Full Time	111	(91.0)	11	(9.0)	
Part Time	4	(100.0)	0	(0.0)	0.39 (p = 1.000)
Work Setting					
Tertiary	47	(87.0)	7	(13.0)	
Secondary	67	(94.4)	4	(5.6)	2.05 (p = 0.205)
Ergonomic Training					
Yes	63	(96.9)	7	(13.0)	
No	44	(93.6)	3	(6.4)	0.69 (p = 0.648)
Postgraduate Training					
Yes	29	(90.6)	3	(9.4)	
No	86	(91.5)	8	(8.5)	0.02 (p = 1.000)

WRMDs: Work-Related Musculoskeletal Disorders

BMI: Body Mass Index

PT: Physical Therapy

*: indicates significance between group differences in the observed proportions for each variable.

### Onset of Disorder

**Figure 1:**
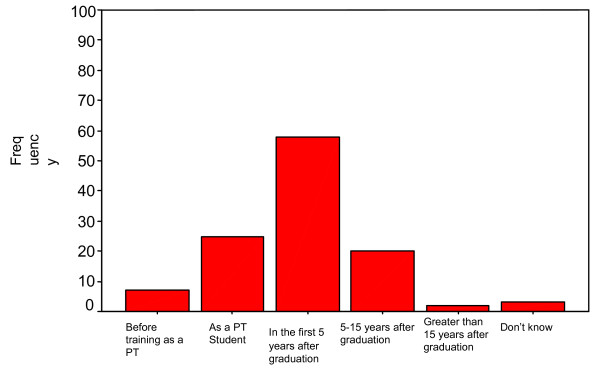
Onset of work related musculoskeletal disorders among participants.

Fifty-eight (46.0%) of the respondents first experienced their WRMDs within the first five years of graduation, while only 2 (1.6%) had it more than 15 years after graduation (Figure 1). The onset of WRMDs was gradual in 83 (65.9%) of the respondents, sudden in 30 (23.8%) and resulted from an accident in 2 (1.6%). (Data not shown).

### Effects of WRMDs

Seventy two (62.6%) of the physiotherapists have changed/modified their treatment for the patients as a result of WRMDs, but 99 (88.4%) did not change their area of practice and 94 (87.0%) did not leave the profession due to their WRMDs. (Data not shown).

### Work Factors

Respondents with WRMDs were asked to consider 17 work factors that have been identified by previous studies [11,12] and indicate the extent to which each contributed to the occurrence of their WRMDs. A likert scale ranging from 1 (indicating not important) to 4 (indicating major importance) was used to ascertain which job risk factor was important in the occurrence of WRMDs. For each work factor the responses were dichotomized into categories of 'not important' (1 and 2) and 'important' (3 and 4). Results were obtained by expressing important responses as percentages of the total responses for each work factor. Eight of the work factors were chosen by 50% or more of the respondents as being important. The two most important work factors commonly identified by physiotherapists were treating large number of patients in a day (83.5%), and working in the same position for long (71.3%). Working with confused or agitated patients (28.9%) and reaching or working away from the body (17.4%) were cited as the most unimportant work factors (Table 4).

**Table 4:** Work factors that physiotherapists identified as contributors to WRMDs

Risks	Number of Respondents	(%)
Treating large number of patients in a day	96/115	(83.5)
Working in same position for long	82/115	(71.3)
Lifting/transferring dependent patients	78/115	(67.8)
Performing manual orthopaedic techniques	78/115	(67.8)
Working in awkward or cramped position	73/113*	(64.6)
Bending or twisting your back in awkward way	72/115	(62.6)
Not having enough rest break during the day	71/115	(61.7)
Carrying, lifting or moving heavy materials or equipments	64/115	(55.7)
Continuing to work when injured	60/115	(52.2)
Performing same task over	60/115	(52.2)
Working at or near your physical limits	54/115	(46.9)
Unanticipated sudden movement or fall by patients	47/115	(40.9)
Work scheduling (overtime, irregular shift, length of workday)	5/114*	(3.5)
Assisting patients during gait activities	41/115	(35.7)
Inadequate training in injury prevention	34/115	(29.6)
Working with confused or agitated patients	33/114*	(28.9)
Reaching or working away from the body	20/115	(17.4)

*- Missing value due to no response by participants

### Coping Strategies

The coping strategies adopted by physiotherapists with WRMDs in Nigeria are shown in Table 5. The two most commonly adopted coping strategies were therapists modifying their positions or the positions of their patients (64.3%) and selecting techniques that will not aggravate or provoke their discomfort (47.0%). The two least adopted coping strategies were the use of electrotherapy instead of manual therapy (9.6%) and warming up and stretching before performing manual technique (5.2%).

**Table 5:** Coping strategies used by physiotherapists with WRMDs.

Strategies	Percentages							
	
	Almost always		Sometimes		Almost Never		No Response	
				
	Number	(%)	Number	(%)	Number	(%)	Number	(%)
I modify patient's position/my position	74/115	(64.3)	31/115	(27.0)	3/115	(2.6)	7/115	(6.1)
I select techniques that will not aggravate or provoke my discomfort	54/115	(47.0)	38/115	(33.0)	15/115	(3.0)	8/115	(7.0)
I adjust plinth/bed height before treating a patient	45/115	(39.1)	35/115	(30.4)	27/115	(23.5)	8/115	(7.0)
I pause regularly so I can stretch and change posture	43/115	(37.4)	44/115	(38.3)	22/115	(19.1)	6/115	(5.2)
I get someone else to help me handle a heavy patient	38/115	(33.1)	50/115	(43.5)	22/115	(19.1)	5/115	(4.3)
I stop treatment if it causes or aggravate my discomfort	28/115	(24.3)	50/115	(43.5)	29/115	(25.2)	8/115	(7.0)
I use different part of my body to administer a manual technique	19/115	(16.5)	39/115	(33.9)	49/115	(42.6)	8/115	(7.0)
I use electrotherapy instead of manual techniques to avoid stressing an injury	11/115	(9.6)	45/115	(39.1)	51/115	(44.3)	8/115	(7.0)
I warm up and stretch before performing a manual technique	6/115	(5.2)	27/115	(23.5)	75/115	(65.2)	7/115	(6.1)

## Discussion

The aim of this study was to investigate the 12- month prevalence and work factors of work related musculoskeletal disorders (WRMDs) among physiotherapists in Nigeria. The percentage response for this study was 58.1% which is consistent with responses in similar studies from Turkey [7] (59%) and Australia [14] (53%) but lower than the 74% reported by Glover et al [13] in the United Kingdom and the 80% by Bork et al [5] in the United States of America (USA). Although, Glover et al [13] utilized the effect of incentives and the influence of the professional association to maximize the response to their study, the relative lower response in our study when compared to others may suggest a lukewarm predisposition to research participation among physiotherapists in Nigeria.

Our finding that there were more male than female physiotherapists in the survey is a reflection of the population from which our sample was drawn. This finding is contrary to the findings from previous studies that reflected more female than male physiotherapists [5-8,13,14]. This result is understandable since unlike in Europe and America, the physiotherapy profession in Nigeria is male dominated. Indeed, 62.3% of the registered physiotherapists in Nigeria are males [23]. The gender distribution of the respondents in our study is hence largely representative of the population of physiotherapists in Nigeria.

We observed a significantly higher prevalence of WRMDs among female physiotherapists with all the female physiotherapists in comparison to 86.3% of the males reporting WRMDs. Our finding is consistent with findings from previous related studies [5,7,13]. Borke et al [5] implicated the female gender as a potential risk factor for the occurrence of WRMDs while Glover et al [13] reported a higher prevalence of work related low back pain, neck pain, shoulder pain and wrist/hand pain among female physiotherapists. A higher but not statistically significant prevalence of WRMDs has also been reported among female Turkish physiotherapists [7]. Cromie et al [8] however reported a higher prevalence of WRMDs among male physiotherapists and attributed their finding to a greater usage of mobilizations and manipulations by male physiotherapists than their female counterparts in their study. It has been suggested that the usually higher prevalence of WRMDs in female physiotherapists may be related to their height and body weight which put them at a disadvantage during patients' treatment and/or transfer [5]. Also, women do have a higher prevalence than men for many upper extremity musculoskeletal disorders, even after controlling for cofounders such as age or work factors [24]. It is interesting however that the prevalence of WRMDs in our study was higher in individuals with normal body weight (94.6%) than obese ones (71.4%).

The 12- month prevalence of WRMDs among Nigerian physiotherapists was found to be 91.3%. This prevalence is higher than the 12-month prevalence of 58% reported by Glover et al [13], 40% by West and Gardner [14], 61% by Bork et al [5] and 62.5% by Cromie et al [8]. The only comparable findings in the literature were the life time career prevalence of 91% and 85% reported by Cromie et al [8] and Salik and Ozcan [7], respectively. The higher 12-month prevalence found in our study suggests that physiotherapy practice in Nigeria highly predisposes to WRMDs. This may be a reflection of the conditions under which physiotherapists practice in Nigeria. Physiotherapy practice in Nigeria, like in many other developing countries is largely bedeviled by unwholesome work settings, understaffing and lack of appropriate equipments including those as basic as standard plinths. This is beside the influence of peculiar cultural values of physiotherapists such as skills, relationships with patients and attitudes of caring and working hard that have been opined as making it difficult for physiotherapists to do their job in a way that minimizes the risk of WRMDs [18]. It should be noted however that the extent to which work contributed to the etiology of musculoskeletal disorders in participants in our study cannot be readily ascertained and is hence largely debatable. It is plausible that some of the physiotherapists in our study probably misconstrued all musculoskeletal disorders as WRMDs regardless of whether these were caused by work or not since the consequence of musculoskeletal disorders and WRMDs in terms of work absenteeism may be similar.

In this study, the low back was reported as the most common site of WRMDs among Nigerian physiotherapists, with a 12- month prevalence of 69.8%. Internationally, the prevalence of work-related low back pain ranged between 22% and 74% [8,13,25]. Our finding is consistent with those of previous studies that have overwhelmingly implicated low back as the body part most commonly affected by WRMDs among physiotherapists [5,7-9,13,14]. In the United Kingdom, the 12-month prevalence of work-related low back pain among physiotherapists was found to be 22% [13], while the prevalence varied between 22% [14] and 62.5% [8] in Australia. Bork et al [5] found the annual prevalence of WRMDs low back pain to be 45% in the U.S.A. Our finding may be a further reflection of the overall picture of the poor conditions of practice that may cause high prevalence of WRMDs among Nigerian physiotherapists.

The majority of the physiotherapists in this study were found to have experienced their first episode of WRMDs within five years of graduation. This is similar to the findings of the majority of studies on WRMDs among physiotherapists [5,6,8,9,13,14]. We also observed the prevalence of WRMDs to be higher among physiotherapists that were younger than 30 years of age. This finding is consistent with those of Salisk and Ozcka [7] in Turkey, Glover et al [13] in the United Kingdom, West and Gardner [14] in Australia, Mierzejewski and Kumar [9] in Canada and Bork et al [5] in the United States of a higher prevalence of WRMDs among physiotherapists younger than 30 years of age. Our finding in this regard is particularly important when viewed against the background of a higher mean age at graduation of physiotherapists in Nigeria compared to their counterparts in Europe, USA and Australia. Our finding hence suggests that physiotherapists in Nigeria may enjoy a shorter WRMD-free career life than their counterparts in other parts of the world. However, findings relating to the onset of injury need to be viewed with caution as it may be very difficult to ascertain the onset of WRMDs without a substantial recall bias.

The work factors commonly identified by physiotherapists in this study as contributing to the occurrence of their WRMDs in decreasing order of importance were: treating a large number of patients in one day, working in the same position for long and lifting or transferring dependent patients, and performing manual therapy techniques. Previous studies have similarly identified treating large number of patients in a day and working in the same position for long periods of time [5,8,14], lifting or transferring dependent patients [6-8,16] and performing manual therapy techniques [14] as the work factors most commonly found to cause WRMDs among physiotherapists. In our study, physiotherapists selected reaching or working away from the body and working with confused or agitated patients as the least important work factors to the occurrence of their WRMDs. It should however be noted that the work factors identified in our study were not specific to individual musculoskeletal disorder but rather cut across various musculoskeletal disorders. This is an important limitation of our study given that previous related studies have submitted that work factors are to some extent specific to individual musculoskeletal disorders. Thus, mobilization and manipulation have been identified as work factors to the occurrence of upper limb, neck, and upper back pain [8]; while performing the same task over and over [13] and lifting and transferring dependent patients [14] have been reported to be related to the occurrence of low back symptoms However, since physiotherapists in our study self- reported the work factors, their responses might have been a reflection of their belief rather than the actual contributions of the work factors to their disorder.

The most commonly adopted coping strategies among physiotherapists in our study were therapists modifying their position or the position of their patients, therapists selecting techniques that will not aggravate or provoke their discomfort, and therapists adjusting bed or plinth height. This finding is similar to that of Glover et al [13], which reported the four most important preventive strategies commonly adopted by physiotherapists in response to sustaining musculoskeletal disorder at work as: therapists adjusting plinth or bed height, therapists modifying their position or the position of their patients, obtaining assistance when handling heavy patients, and ceasing a patient's treatment if such treatment aggravates or provokes their symptoms. Further, most of the physiotherapists in our study would also change or modify a patient treatment in the face of their WRMDs thus suggesting that physiotherapists in Nigeria who had experienced WRMDs might have sometimes selected treatment methods for reasons other than the needs of their patients – their own comfort. This attitude may not augur well for the application of the principle of altruistic care needed for effective patient treatment and optimal recovery.

Despite the high prevalence of WRMDs among Nigerian physiotherapists, we found that the majority of the physiotherapists did not leave the profession and only a few changed their area of practice/specialty. Our finding is consistent with those of majority of studies that found that few physiotherapists will change their areas of practice [7,8,13] and majority will not leave the profession [5,8,9,13,14] as a result of WRMDs. While previous studies which were conducted mostly in developed countries explained their findings in the context of 'survival bias' developed during career [5,14], adaptation to injury [13], flexibility of work change within profession [8,14] and the culture of physical therapy [18], our finding may be suggestive of limited career-change option among Nigerian physiotherapists. This view is buttressed by our finding that 96.5% of Nigerian physiotherapists work full time and that only about a quarter had postgraduate training- an option which might have enhanced their choice of area of practice/specialty. Further, the economic vagaries and palpable financial insecurity in Nigeria may actually make physiotherapists in Nigeria to stay put within the profession despite its attendant high risk of WRMDs. The real reason or reasons why Nigerian physiotherapists do not leave the profession in spite of the high rate of WRMDs would however need to be further investigated by future studies.

### Limitations

This study is limited by the sampling technique employed, as the non-probability sampling employed in our study may prevent generalization of our results. We could not randomize because the list of registered physiotherapists in Nigeria as contained in the Nigerian Medical Rehabilitation Therapists Bulletin [19] did not reflect the addresses and workplaces of registered members. We however tried to minimize this effect by administering our survey in all the 26 accredited tertiary and secondary health institutions in the six geopolitical zones and the federal capital territory of Nigeria, in the hope that our sample will reflect the geopolitical diversity and heterogeneity of Nigeria.

Like all other cross-sectional studies involving recall, our respondents might have given vague answers to questions asked in this study as they might not have remembered the information requested of them easily. In an attempt to curtail the influence of this in our study, we restricted our survey to a 12-month prevalence which would have tasked the participants' memory lesser than the conventional lifetime and career prevalence. We however defined WRMDs as any pain or discomfort that lasted more than three days in the last 12 months in the hope that the respondents would be able to remember significant periods of their discomfort. We equally appreciate that work may only be a contributory factor in the etiology of musculoskeletal disorders among workers and that it may be difficult to distinguish between WRMDs and musculoskeletal disorders since their consequences in response to work demands may be similar. It is thus plausible that some of the respondents in our study perceived their musculoskeletal disorders as WRMDs regardless of whether they were caused by work or not.

Despite these limitations, our study has provided for the first time data on the prevalence and work factors of work-related musculoskeletal disorders among physiotherapists in Nigeria. It has also underscored the need for further studies on the behavioural consequences of WRMDs and career attitudes of Nigerian Physiotherapists to them.

## Conclusion

This study reveals that the 12-month prevalence of WRMDs among physiotherapists in Nigeria is higher than most values reported for their counterparts around the world, but reflected similar work risk factors and coping strategies. Further studies on the consequences of WRMDs and why Nigerian physiotherapists remain in the profession despite the inherent high prevalence of WRMDs are imperative and hence suggested.

## Competing interests

The authors declare that they have no competing interests.

## Authors' contributions

BOA conceptualized and designed the study, was involved in data interpretation and read the final manuscript. AK was involved in data acquisition and drafting of the manuscript. AL analyzed the data and revised the manuscript for important intellectual content. All authors read and approved the final manuscript.

## Pre-publication history

The pre-publication history for this paper can be accessed here:



## Supplementary Material

Additional file 1 Occupational Health and the Practice of Physiotherapy Questionnaire. Click here for file
