# Supplementary material for: Work-related musculoskeletal disorders among Nigerian Physiotherapists
Source: BMC Musculoskelet Disord. 2008 Aug 18;9:112. doi: 10.1186/1471-2474-9-112 (PMC2535595; doi:10.1186/1471-2474-9-112)
Supplement: Additional file 1 — Occupational Health and the Practice of Physiotherapy Questionnaire. [file 1471-2474-9-112-S1.doc]

**Questionnaire: Re: Occupational Health and the Practice of Physiotherapy**

**SECTION A.**

1. **Age (as at last birthday)________________________________**
2. **Height (m)___________________________________________**
3. **Weight (kg)__________________________________________**
4. **Gender- Female____________**

**Male______________**

1. **(a) Year of Graduation from physiotherapy school_________________**

**(b) Year of Professional experience______________________________**

1. **School of physiotherapy from which qualification was obtained__________________________________________________**
2. **Postgraduate qualification (s)- Yes__________**

**No__________**

**8. Work Status in the Last 12 Months- Full Time___________**

**Part Time___________**

1. **(a) In what town/city and state do you currently practice_______________________________________**
   1. **___________________________________ region/province**
2. **(a)Please indicate your work setting- Tertiary___________________**

**Secondary__________________**

**(b)Please give the approximate hours per week (HPW) you spend in direct patient care in the last 12 month as part of your practice of physiotherapy____________________________________________**

**SECTION B.**

**11. Have you ever experienced work-related pain or discomfort in any part of you body that lasted for more than 3 days in the last 12 months?- Yes________________**

**No_________________**

1. **If you answered yes to Q11, consider the most significant work- related problem you have experienced and indicate the location?**
   - **Neck?_______________**
   - **Shoulders?________________**
   - **Upper Back?________________**
   - **Elbow/forearm?_____________**
   - **Low back?__________________**
   - **Wrists/hands?_______________**
   - **Thumbs?___________________**
   - **Hips/thighs?_________________**
   - **Knees?_____________________**
   - **Ankles/feet?_________________**
2. **When did you first experienced this work-related pain or discomfort**

**- Before training as a physiotherapist?_________________________**

**- As a physiotherapy student?_________________________________**

**- In the first 5 years after graduation?__________________________**

**- 5-15 years after graduation?_________________________________**

**- >15 years after graduation?_________________________________**

**Don’t’ know?_____________________________________________**

1. **Was the onset:**
   - **Gradual?______________**
   - **Sudden?_______________**
   - **As a result of accident?___________**
2. **Have you ever changed or modified treatment as a result of work-related discomfort?**
   - **Yes_________________**
   - **No__________________**
3. **Have you changed the area/ specialty in which you practice as a result of work-related discomfort?**
   - **Yes__________________**
   - **No___________________**
4. **If the answer is yes, what did you change from?**
   - **From______________________________**
   - **And to______________________________**
5. **Have you ever had training in ways to alter your environment to reduce strain on your body (ergonomics)?**
   - **Yes_____________**
   - **No______________**
6. **Which of the following do you use to reduce to reduce the strain on your body when working?**
   - **Adjustable bed/plinth_____________**
   - **Sliding board____________________**
   - **Patient lifting belt________________**
   - **Splint__________________________**
   - **Other__________________________**
   - **None of the above________________**
7. **Which of these conditions have you had a result of your work as a physiotherapist?**
   - **Hearing loss_____________________**
   - **Fungal skin infection______________**
   - **Bacterial skin infection_____________**
   - **Asthma__________________________**
   - **Dermatitis/skin rash_______________**
   - **Hepatitis B_______________________**
   - **HIV/AIDS________________________**
   - **TB______________________________**
   - **Others___________________________**
   - **None of the above__________________**
8. **Have you had any of these conditions (not necessarily related to your work as a physiotherapist)?**
   - **Cataract (s)____________________________________**
   - **Skin lesion (Type……………………)_________________**
   - **Heart disease_____________________________________**
   - **Malignancy________________________________________**
9. **Have you left the physiotherapy profession to pursue another career as a result of work related disorder**
   - **Yes and permanently___________________**
   - **Yes and temporarily____________________**
   - **No___________________________________**

**Job Risk factors**

1. This list describes factors that could contribute to work related discomfort or injury. In your opinion, how have the following factors contributed to your work-related discomfort or injury?

| **s/no** | **Risk factors** | **Irrelevant**  **(1)** | **Minor or insignificant way (2)** | **Moderately significant**  **(3)** | **Major Significant way (4)** |
| --- | --- | --- | --- | --- | --- |
| **1** | **Performing the same task over and over** |  |  |  |  |
| **2** | **Treating a large number of patients in one day** |  |  |  |  |
| **3** | **Not enough rest breaks during the day** |  |  |  |  |
| **4** | **Performing manual orthopaedic techniques (joint or soft tissue mobilization)** |  |  |  |  |
| **5** | **Working in awkward or cramped positions** |  |  |  |  |
| **6** | **Working in the same position for long periods (standing, bend over, sitting, etc)** |  |  |  |  |
| **7** | **Bending or twisting your back in an awkward way.** |  |  |  |  |
| **8** | **Reaching or working away from your body** |  |  |  |  |
| **9** | **Unanticipated sudden movement or falls by patient** |  |  |  |  |
| **10** | **Assisting patient during gait activities** |  |  |  |  |
| **11** | **Lifting or transferring dependent patients** |  |  |  |  |
| **12** | **Working with confused or agitated patients** |  |  |  |  |
| **13** | **Carrying, lifting or moving heavy materials or equipment** |  |  |  |  |
| **14** | **Working at or near your physical limits** |  |  |  |  |
| **15** | **Continuing to work when injured or hurt.** |  |  |  |  |
| **16** | **Work scheduling (over time, irregular shift, length of workday)** |  |  |  |  |
| **17** | **Inadequate training in injury prevention.** |  |  |  |  |

**Coping Strategies:** The response to the following statements should reflect what you actually do in practice rather than what you would like to do or think you should do.

1. In order to reduce the strain on my body when working

| s/no | Strategies | Almost Always | Sometimes | Almost Never |
| --- | --- | --- | --- | --- |
| 1 | **I get someone else to help me handle a heavy patient** |  |  |  |
| 2 | **I modify patient’s position/ my position** |  |  |  |
| 3 | **I use a different part of my body to administer a manual technique** |  |  |  |
| 4 | **I warm up and stretch before performing manual technique.** |  |  |  |
| 5 | **I use electrotherapy instead of manual techniques to avoid stressing an injury** |  |  |  |
| 6 | **I pause regularly so I can stretch and change posture.** |  |  |  |
| 7 | **I adjust plinth/bed height before treating a patient.** |  |  |  |
| 8 | **I select techniques that will not aggravate or provoke my discomfort.** |  |  |  |
| 9 | **I stop a treatment if it causes or aggravate my discomfort** |  |  |  |

1. The type of patient I usually treat

| s/no | Patients | Irrelevant | Almost Always | Sometimes | Almost Never |
| --- | --- | --- | --- | --- | --- |
| 1 | Requires minimal or no assistance |  |  |  |  |
| 2 | Requires moderate assistance (1 person to assist) |  |  |  |  |
| 3 | Requires maximum assistance (2 people to assist) |  |  |  |  |

1. Can you suggest any ways in which you could better prepare for work as a physiotherapist in terms of looking after your health?

________________________________________________________________________________________________________________________________________________________________________________________________________________________

1. Have you any other comments?

________________________________________________________________________________________________________________________________________________________________________________________________________________________

Thank you for you assistance.
